# Understanding molecular characteristics of extracellular vesicles derived from different types of mesenchymal stem cells for therapeutic translation

**DOI:** 10.1016/j.vesic.2024.100034

**Published:** 2024-03-02

**Authors:** Zuo Ding, Zachary F. Greenberg, Maria Fernanda Serafim, Samantha Ali, Julia C. Jamieson, Dmitry O. Traktuev, Keith March, Mei He

**Affiliations:** a Department of Pharmaceutics, College of Pharmacy, University of Florida, Gainesville, FL, 32611, USA; b UF Center for Regenerative Medicine, Division of Cardiovascular Medicine, Department of Medicine, College of Medicine, University of Florida, Gainesville, FL, 32610, USA

**Keywords:** Extracellular Vesicles, Mesenchymal Stem Cells, Molecular Characteristics, Therapeutic Translation

## Abstract

Mesenchymal stem cells (MSCs) have been studied for decades as candidates for cellular therapy, and their secretome, including secreted extracellular vesicles (EVs), has been identified to contribute significantly to regenerative and reparative functions. Emerging evidence has suggested that MSC-EVs alone, could be used as therapeutics that emulate the biological function of MSCs. However, just as with MSCs, MSC-EVs have been shown to vary in composition, depending on the tissue source of the MSCs as well as the protocols employed in culturing the MSCs and obtaining the EVs. Therefore, the importance of careful choice of cell sources and culture environments is receiving increasing attention. Many factors contribute to the therapeutic potential of MSC-EVs, including the source tissue, isolation technique, and culturing conditions. This review illustrates the molecular landscape of EVs derived from different types of MSC cells along with culture strategies. A thorough analysis of publicly available omic datasets was performed to advance the precision understanding of MSC-EVs with unique tissue source-dependent molecular characteristics. The tissue-specific protein and miRNA-driven Reactome ontology analysis was used to reveal distinct patterns of top Reactome ontology pathways across adipose, bone marrow, and umbilical MSC-EVs. Moreover, a meta-analysis assisted by an AI technique was used to analyze the published literature, providing insights into the therapeutic translation of MSC-EVs based on their source tissues.

## 1. Introduction

Due to regenerative and immune-modulating functions shown in both *in vitro* and *in vivo* studies, mesenchymal stem cells (MSCs) have been recognized as potentially important therapeutic agents for clinical translation. MSCs are a type of self-renewing and multipotent progenitor cell,^1^ and have been successfully isolated and cultured from various organs, including bone marrow,^2,3^ umbilical cord,^4^ adipose tissue,^5^ and many other vascularized tissues.^6^ As of December 2023, more than 1500 clinical trials related to MSCs have been registered (clinicaltrials.gov, “mesenchymal stem cell”). Approximately 10 MSCs products have been approved worldwide (i.e. Europe, Canada, New Zealand, Japan, South Korea and India) for a few conditions including graft-versus-host disease (GvHD), Crohn’s disease (CD), and amyotrophic lateral sclerosis (ALS).^7^ However, in the United States, the U.S. Food and Drug Administration (FDA) has not yet approved an MSCs product for commercialization. Important impediments to such approvals include concerns relating to reproducibility of cell product identity and potency.

Growing evidence has supported the concept that the tissue repair and immunosuppressive functions of MSCs are largely due to their paracrine secretion of multiple bioactive factors, as we first proposed nearly 20 years ago^8^; these factors include extracellular vesicles (EVs).^9–12^ EVs, especially exosomes, are lipid-bilayer structured, nano-size particles which are secreted by nearly all living cell types as a means of intercellular communication.^13,14^ EVs can carry various types of proteins, lipids, RNAs and DNAs which can effectuate many of their parent cellular functions, and accordingly EVs have been suggested as cell-free alternative therapeutic agents.^15,16^ MSC-EVs have demonstrated biological impacts comparable to their parent cells in multiple models of human disease, communicating the regenerative and anti-inflammatory properties of MSCs.^17,18^ The MSC-EVs in particular provide a more practical handling and therapeutic administration than their parent cells, in part by eliminating the need for maintaining cryogenic temperatures during the post-production supply chain. MSC-EVs also maintain a more stable phenotype, once generated, in comparison to MSCs’ potential for alteration during culture passage.^19^ Additionally, though MSCs were once thought to be immune-privileged, allogeneic MSCs can trigger anti-donor immune responses.^20,21^ In contrast, MSC-EVs appear less immunogenic, which provides a viable alternative to reduce immune rejections.^22,23^ Accordingly, the clinical application of MSC-EVs has been emerging as an alternative allogeneic therapy. As of early 2023, there were 39 clinical trials registered on the clinicaltrials.gov, evaluating MSC-EVs as therapeutics. In considering these trials, it is important to recognize that since FDA authorization is not included as a criterion for determining whether or not a trial can be registered on that website, many of these may not be proceeding under an FDA-authorized Investigational New Drug (IND) or Investigational Device Exemption (IDE).^24^ Due to the outbreak of COVID-19 in 2020 and the lack of effective therapeutics in the early stage, many clinical trials proposed to apply MSC-EVs into the treatment of COVID-19 related complications, taking advantage of the immunomodulating and regenerative function of MSC-EVs. Other clinical applications of MSC-EVs often involve the repair of injured tissues and regulation of immune response in certain conditions such as transplant rejection.^18^ However, regulatory agencies around the world have not yet approved any MSC-EVs related therapeutics for general use. More studies are needed to understand and prove the therapeutic efficacy of MSC-EVs.

Although MSC-EVs have drawn much attention in regenerative medicine, as noted above, MSC-EVs display their own unique properties due to different tissues of origin and culture conditions. In order to precisely translate EVs for clinical applications, understanding MSCs tissue sources relevant to EV production and associated molecular components will be profoundly important. Herein, we reviewed relevant studies on MSC-EVs in the last 10 years using meta-analysis, which investigates omic profiles from MSC-EVs in terms of tissue-specific protein and miRNAs. Via Reactome ontology analysis, the result revealed distinct patterns of top Reactome ontology pathways across adipose, bone marrow, and umbilical EVs. Using reported omic datasets, the unique molecular component characteristics can be identified to define different types of MSC-EVs, which in turn may enhance the foundation for clinical applications through the careful choice of MSC-EVs.

## 2. Sources of mesenchymal stem cells

MSCs can be obtained from many different tissues of the human body, with the most popular being bone marrow, adipose tissue, and umbilical cord. According to FDA, prior to 2007, 100% of MSCs-related INDs used bone marrow (BM) as the source tissue; however, by 2012, only about half of INDs were collecting MSCs from bone marrow, while umbilical cord (UC) and adipose tissue (AT) became the second and third most frequently employed source tissues, respectively.^25^ Additionally, more recent studies have used MSCs isolated from other tissues, including articular cartilage, brain, dental pulp, skin, blood, and amniotic fluid.^26^

**Fig. 1.**
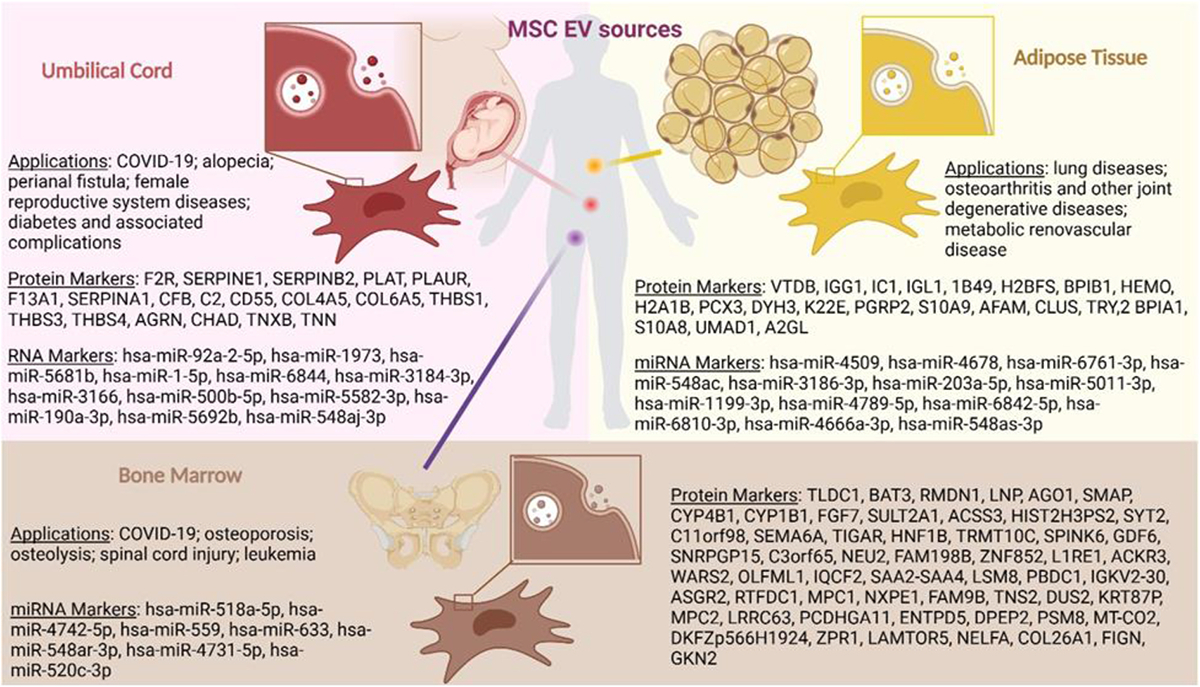
Source tissues of MSC. Applications of each EV category were identified and summarized from literature. Protein and miRNA markers were screened using *meta*-proteomic and transcriptomic analysis of reported omic datasets.

We gathered reported literature regarding their EV omic datasets to establish a new ExoMEGA database. The *meta*-proteomic and transcriptomic analysis was used to identify protein and miRNA markers based on unique expressions in respective source tissue. In total, 21 adipose protein markers and 56 bone marrow protein markers were identified and listed in Fig. 1. Due to the large number of protein markers identified for umbilical cord (n = 1393), the most significant markers were selected as proteins that are involved in signaling pathways indicated from studies associated with the deposited datasets. The analysis was carried out by use of the STRING bioinformatic tool.^27^ By this means, a total of 19 protein markers were identified, including some related to coagulation^28,29^ and ECM-receptor interaction.^29–31^ A similar approach was employed to identify significant miRNA markers for BM-MSCs (n = 134), AT-MSCs (n = 689) and UC-MSCs (n = 94) derived EVs with miRPathDB v2.0. This tool suggested that BM-MSC-EVs harbor prominent functions in anti-apoptosis^32^ and astrocyte differentiation.^33^ AT-MSC-EVs showed strong effects in phagocytosis,^33,34^ cell motility^34^ and osteoblast differentiation.^35^ In addition, UC-MSC-EVs are reported to be involved in angiogenesis,^33^ improvement of spinal cord injury,^36^ and regulation of various signaling pathways, including toll-like signaling pathway,^37^ IL-17 signaling pathway,^37^ and Transforming Growth Factor-beta (TGF-beta) signaling pathway.^38^

### 2.1. Bone marrow

Bone marrow is a soft tissue located in the bone cavity for generating blood cells, which houses two different populations of stem cells, hematopoietic stem cells (HSCs) and MSCs.^39^ Early studies suggested that bone marrow mesenchymal stem cells (BM-MSCs) were at least tri-potent and could differentiate into adipocytes, osteoblasts and chondrocytes.^40^ In distinction from differentiation potential, the regenerative and immunomodulating functions of BM-MSCs are also carried out by its paracrine secretion of proteins such as growth factors and cytokines, as well as EVs that carry many bioactive molecules.^10,11^ Proteome analysis of the secretome including EVs from human BM-MSCs has revealed cytokines, growth factors and essential proteins regulating hematopoiesis, including vascular endothelial growth factor C (VEGF-C), TGF-β and growth differentiation factor 6 (GDF6).^41,42^ Secretome analyses of human BM-MSCs also identified VEGF-A, Angiopoietins (ANGPTs), insulin-like growth factor-1 (IGF-1) and hepatocyte growth factor (HGF), which play roles in pro-survival, angiogenesis^43^, and bone regeneration^44^.

### 2.2. Umbilical cord

Umbilical cord mesenchymal stem cells (UC-MSCs) are mostly derived from Wharton’s jelly or the lining of the umbilical cord. UC-MSCs can be isolated and retrieved with no ethical or legal considerations, because they are obtained after parturition and are regarded as waste byproducts; their procurement does not affect the infant.^45^ UC-MSCs also have the ability to secrete numerous growth factors, cytokines, adhesion molecules, chemokines, and associated EVs, which can promote physiological functions like cell migration, MSCs migration, angiogenesis, wound healing, anti-apoptosis, neuroprotective, anti-inflammation, and pro-inflammation among others.^46^ UC-MSC-EVs are reported to be highly abundant^47^ and express membrane-bound proteins found in mesenchymal cells such as positive expression of CD90, CD105, CD44 and negative CD11b, CD34, CD45^48^, which could potentially imply that they possess properties and therapeutic function simulating those of the parent cells^49,50^. Moreover, it has been reported that MSC-EVs mediated therapies can circumvent issues like necrosis, dysregulated differentiation, and immune rejections caused by cell transplantation^51^, making them attractive for potential use in cell-free clinical applications.

### 2.3. Adipose tissue

Adipose tissue, also known as body fat tissue, is distributed throughout the body under the skin, surrounding organs in the abdominal cavity, as well as in the bone cavity as an important part of bone marrow.^52^ Similar to BM-MSCs, adipose tissue derived MSCs (AT-MSCs) are also multipotent and can differentiate into adipocyte, osteoblast and chondrocyte.^53^ However, compared to BM-MSCs, AT-MSCs are superior in adipogenesis, while their ability of chondrogenesis and osteogenesis is lower.^54,55^ Similar to BM- and UC-MSCs, AT-MSCs secretome includes many regeneration-facilitating factors, as we have reported in multiple studies since our first report in 2004,^56^ including EVs. AT-MSC-EVs are capable of regulating adipocyte functions and providing control of obesity-related complications.^57^ In addition, proteomic analysis of human AT-MSC-EVs has identified a number of proteins involved in proliferation-regulating PI3K-AKT, JAK-STAT and Wnt signaling pathways, and showed for improving urethral functions in rats.^58^

### 2.4. Other tissues

In addition to the most popular tissue sources discussed above, many other tissues have emerged to serve as origins for MSCs. Dental pulp (DP) locates in the core of a tooth consisting of nerves, blood vessels and soft tissues where MSCs reside. Due to their unique origin, DP-MSC-EVs are believed to serve greater roles in dental and neurological diseases compared to EVs of other origins.^59^ Notably, a comparative analysis of BM-MSC-EVs and DP-MSC-EVs identified 21 differentially expressed PIWI-interacting RNAs (piRNAs) that contribute to biological functions of MSC.^60^

Although peripheral blood is relatively easier to obtain than bone marrow, the population of MSCs among peripheral blood cells is significantly less,.^61^ rendering blood a little-employed source of MSCs. Nontheless, certain populations of cells from peripheral blood are found to present fibroblast-like features; characterization of those cells shows presentation of similar surface biomarkers and multipotency to MSCs.^62^ MSCs with similar characteristics to BM-MSCs are also present in the endometrium and can be obtained from menstrual blood.^63^ Menstrual blood-derived MSCs have shown preclinical efficacy toward a wide variety of diseases through their regenerative and immunoregulating functions.^64^

Perinatal MSCs are derived from tissues and fluids associated with childbirth. In addition to umbilical cord tissue, these sources include cord blood, placental tissue, and amniotic fluid. Compared to adult MSCs, perinatal MSCs appear to pose advantages of better proliferative capacity and lower immunogenicity^65^. In addition, as with UC-MSC, other perinatal MSCs are easily obtained from tissues that are considered medical waste and there is very little ethical issue associated, rendering them highly attractive. MSCs derived from amniotic membrane and fluid, and chorionic plate present fetal features, while cells derived from decidua parietalis are adult MSCs from the mother^66,67^.

## 3. MSCs culture and expansion

### 3.1. MSCs culture conditions

One of the main concerns regarding appropriate conditions for culturing MSCs is the impact on differentiation potential. Estes et al. showed that the differentiation of MSCs is highly susceptible to environmental factors such as initial seeding density, levels of growth factors in the basal media and even plasticware for culture.^68^ The current medium commercialized for different types of MSCs culture falls into one or more of the following categories, which will be discussed in the following order: (1) serum-containing, (2) Good Manufacturing Practices (GMP)-grade, (3) serum-free (SF), (4) xeno-free (XF) and (5) chemically defined.

Traditionally, MSCs have been expanded *ex vivo* under static serum-rich conditions. Serum, however, has raised many controversies due to its inconsistencies between batches and risk of microbiological contamination.^69,70^ The manipulation of MSCs to an albumin-free version should, however, be done very carefully, since MSCs have been found to be more sensitive to even small environmental changes in the absence of albumin.^71^ In turn, fibroblast growth factor 2 (FGF2)’s inconsistencies stem from its thermal instability, leading to precipitation in solution or conformational changes of proteins. Heparin has been used in different studies to stabilize FGF2^72,73^ and in other cases FGF2 has even been mutated into stable forms such as K18 N.^74^ To address ongoing concerns regarding fetal bovine serum (FBS), current studies have been shifting along two major routes: (1) replacing FBS with either allogeneic or autologous human serum derived components or (2) removing serum altogether by designing cell-specific chemically defined medium. A series of different studies have shown that serum-free and Xeno-free media (SF/XFM) are able to either maintain MSCs culture at a level equal to or better than that of serum-rich medium.^69,70,75^ The need for GMP-compliant conditions for clinical applications over recent years has made researchers shift away from traditional MSCs culture conditions and towards xeno/FBS-free chemically defined medium. Though pre-clinical studies have shown MSCs potential in treating a wide range of diseases including neovascularization, cardiac diseases and spinal cord injury, an important bottleneck still lies in translating consistency from bench results to clinical settings, due to lack of GMP-compliant practices. Following GMP quality culture conditions is an essential step preceding clinical trials, which would increase bench-to-clinic turnover in MSCs research.

MSC-EVs are equally affected by variability in culture conditions. Culturing MSCs under GMP-compliant-XF/SFM not only enables production of EVs with conserved classical functional properties but can also enhance EV quantities.^76^ In fact, some studies suggest that XF/SFM may even enhance MSC-EVs’ cardiomyogenic and angiogenic potential.^77^ A comprehensive comparison of the current FBS-free alternatives was done by Oikonomopoulos et al. Their results led to the following conclusions: (1) both human platelet lysate (HPL) and XF/SFM increased the proliferation of MSCs; (2) HPL diminished the immunosuppressive properties for MSCs; (3) BM-MSCs and AT-MSCs in FBS and serum/xeno-free media showed potent immunosuppressive properties when primed with interferon (IFN)-γ; (4) both HPL and SF/XFM primed with IFN-γ increased levels of indoleamine 2,3-dioxygenase 1 (IDO-1) compared to FBS.^78^ These results suggest that SF/XFM conditions are superior to HPL-based culture of MSCs, but further transcriptomic sequencing studies are required to investigate the different methods’ effect on the genomic profiles of the cells. The emerging ongoing trend seeks to replace cell-based therapies with cell-free EV based therapies, prompting further research into the standardization of a universal Xeno-FBS-free culture system for MSCs for clinical applications.

Cross-comparison of studies across the MSCs field is often difficult due to the variety of culture conditions utilized by different researchers. Baer et al. attempted to standardize culture conditions by establishing an expansion medium optimal for maintaining the undifferentiated state of AT-MSCs.^79^ Their results showed that two commercially available media, by PAA Laboratories and StemCell Technologies, respectively, were most appropriate in expansion of AT-MSCs as they resulted in a significant increase of expression of transcription factors and HGF and maintained *ex vivo* MSCs morphology.^79^ Ahearne et al. compared how AT-MSCs cultured with DMEM and DMEM-F12 supplemented with and without FGF differed. They found that addition of growth factors may only be beneficial at the earlier passages for these cells and that high glucose levels supplemented with high levels of FGF and FGF supplemented basal media were better suited for priming cells for keratogenesis and chronogenesis respectively.^80^ We searched the patent scope database (keywords: MSCs, Xeno-free, FBS-free and chemically modified) to summarize current different types of stem cell culture media, which represents MSCs culture status in Table 1. Though many studies have identified culture media as a major factor influencing MSCs differentiation potential, not many studies have looked at optimizing and standardizing medium types for each type of MSCs differentiation. This is particularly important from a manufacturing standpoint as studies progress to clinical phases because it is critical for clinical utility to ensure reproducibility and consistency in results.

Scalability is a key factor in the transition from bench to clinic and coincidently a major bottleneck with stem cell therapy. With the hope of addressing this gap, Devito et al. proposed that oxygen deprivation could increase the number of Wharton’s jelly-derived MSCs positive for MSCs antigen 1 which is a defining characteristic of clinical-grade BM-MSCs.^86^ This could increase the number of clinically useful MSCs, but results pend further investigation to address whether hypoxic conditions promote mutations in future generations of cells. Furthermore, a number of studies were established, demonstrating hypoxia pre-conditioning of MSCs can improve their performance in regenerative functions, partly due to altered secretome including soluble factors and EVs.^87^

### 3.2. 2D vs. 3D culture of MSCs for EV production

**Fig. 2.**
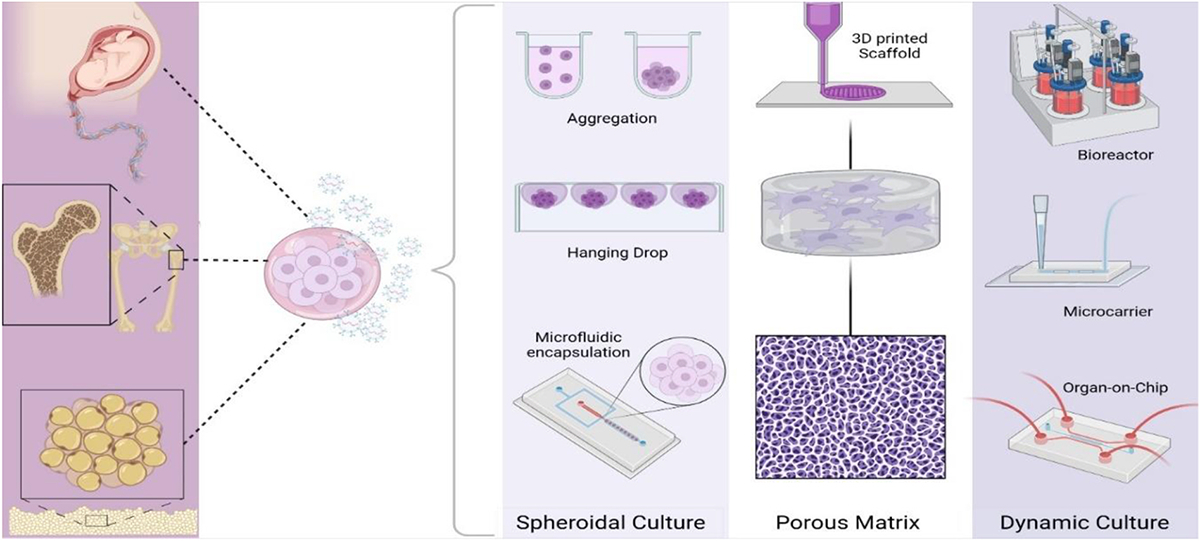
Schematic illustration of 3D culture of MSCs for EV production.

Although being the traditional form of cell culture, growing MSCs in 2D formats can have negative impacts on altered growth kinetics of native MSCs and differentiation potency, as well as triggering premature senescence.^88^ 3D cell culture models possess an arrangement of cells within an extracellular matrix (ECM), which is generally comprised of scaffolds of structural proteins like collagen, alginate, gelatin, fibrin, chitosan, among others, and synthetic polymers like polylactic acid (PLA), polyglycolic acid (PGA), polyurethane (PU), poly lactic-*co*-glycolic acid (PLGA), and polycaprolactone (PCL),^89^ and have been recognized for providing a more realistic translation of in-vivo cell behaviors.^90^ In 3D culture, cells can sense external mechanical stimuli and respond by activating mechanotransduction-related molecular pathways that regulate cell growth, differentiation, adhesion, and signal transduction.^91^ Consequently, the produced EVs could be more representative of *in vivo* physiologically relevant EV secretion, which could substantially improve their therapeutic potential for clinical application. Utilizing 3D culture systems as illustrated in Fig. 2 may accordingly enhance the production and study of EVs. A particular study demonstrated that UC-MSCs cultured in scalable microcarriers-based 3D culture system using serum-free/GMP- compatible medium could yield 20-fold more EVs than 2D cultured UC-MSCs.^92^ In another study, UC-MSCs cultured in a hollow fiber bioreactor not only yielded up to 7.5-fold higher production of EVs compared to 2D culture, but these EVs also exerted stronger effects in chondrocyte proliferation, migration, matrix synthesis, and displayed more prominent therapeutic effects in cartilage defects.^93^ BM-MSCs cultured into spheroids in different sizes and using different biomaterials for encapsulation produced significantly more EVs than those cultured in conventional monolayer cultures.^94^ Table 2 provides an overview of studies performed using MSC-EVs cultured in 3D environments and their outcomes.

### 3.3. Molecular characteristics of various types of MSC-EVs

The isolation process of MSCs from their respective sources is by nature highly heterogeneous. Particularly, impurities that are co-isolated with MSCs during the aspiration, such as the presence of fibroblasts,^100^ pose a daunting challenge regarding the clinical translatability of MSCs and MSC-EVs based therapies. The isolation purity of MSCs serves as a foundational step in obtaining their EVs as they can be collected from MSCs culture media through a variety of techniques such as ultracentrifugation, ultrafiltration, size-exclusion chromatography, or precipitation techniques.^101^

In 2006, the International Society for Cellular Therapy released a position statement outlining the basic requisites defining multipotent MSCs, with the hope of standardizing basic stem cell research protocols to produce comparable results. The statement described that MSCs must express CD105, CD173 and CD90, and lack expression of CD45, CD34, CD14 or CD11b, CD79alpha or CD19 and human leukocyte antigen-DR isotype (HLA-DR) surface molecules.^102^ As expected, MSC-EVs, present not only EV-specific markers like CD81, CD9, CD63 and CD107 but also present the previously described MSC-specific markers like CD73, CD44 and CD90^103 104^. This confers EVs’ wide therapeutic applications including but not limited to anti-senescence,^105^ wound-healing,^106^ various tissue regeneration,^107–109^ and novel cancer therapies^110^ etc. These markers, however, are present at varying abundances, with CD44 being the most prevalent, followed by CD90 and CD73 respectively.^104^ Each marker plays a unique role in the different EV-stimulated regenerative processes, which makes an essential consideration when developing MSC-EV-based therapies.

Though markers are generally seen as broadly applicable, meaning that all EVs released from a particular cell type will be homogeneous in the types of markers they present, the source variability in MSCs prevents this from being true. A study conducted by Gorgun et al. comparing the content of small-to middle-sized EVs from adipose and bone marrow tissues showed that there is a reasonable amount of variation not only between EVs proteomic and genomic profiles based on tissue origin, but also on their surface detection markers.^111^ In fact, adipose-derived MSC-EVs presented with 30% total expression of CD34 which would generally indicate contamination of endothelial, myeloid or hematopoietic cells, but in the case of adipose-derived EVs it is completely normal. Adipose-derived EVs showed significant upregulation of proteins involved in injury modulation (DKK-1, GRO-α, IL-8 AND IGFBP-3), while bone marrow-derived EVs overexpressed proteins involved in osteogenesis and angiogenesis (ANG-2, BDNF, IFN-γ, IL-1α, KLK-3 and RETN).^111^ Additionally, pro-regenerative ability has been observed with MSC-EVs which act by stimulating cell proliferation, inhibiting apoptosis, and favoring immune escape. It is important to understand the unique profile of EVs derived from different sources, due to the previously discussed marker variability, as specific source MSC-EVs may be preferentially applied to certain types of tissue therapies over others. In AT-MSC-EVs, neprilysin also known as CD10, may play a role in restoring nerve tissue often associated with diseases like Alzheimer’s, though this relationship pends further study.^112^ Another study suggests that CD73, a marker specifically expressed by BM-MSC-EVs, is involved in the pathway that leads A2AR-expressing T-helper type 1 (Th1) cells to apoptosis, relevant to immune pathways of the respiratory system.^113^ In renal disease, BM-MSC-EVs presenting CCR2, a C–C motif chemokine receptor, enabled macrophage suppression, alleviating the effects of ischemia/reperfusion-induced renal injury.^114^ The immunomodulatory abilities of MSC-EVs may also be affected by cell passage, as senescence can degrade EVs defining surface markers.^115^ A study comparing MSC-EVS size, yield, and levels of surface markers between passages 5 (P5) and 15 (P15) showed that though yield and size remained constant, the expression of exosomal markers in passage 15 cells, particularly CD9, greatly decreased as compared to passage 5 cells.^115^ Moreover, the study demonstrated that EV’s immunomodulatory abilities were negatively affected by passage, as P15 EVs were less effective than P5 EVs in suppressing the secretion of TH1 and TH17 cytokines as well as stimulating TGF-β production.

Undoubtedly, there needs to exist a uniform protocol to assess the purity of different source MSC-EVs, which means creating a middle ground for markers that are generally present in all MSC-EV types.^116^ In addition to the well-defined MSC-EVs markers, some novel markers have been found in the profiles of all different source MSCs. An adhesion molecule, CD29, is one such marker, which works with CD44 to control the entry of MSC-EVs into target cells.^103^ CD349 or Frizzled-9, which had previously shown great potential in specifically isolating BM-MSCs from human placenta,^117–120^ has also been identified in BM-MSC-EVs as one of the only common makers between different donors.^121^ This is consistent with Tran et al.’s results who not only identified FZD-9 on the surface of different lines of human placenta MSCs, but also pinpointed its potential as an MSC marker indicative of the cells’ reendothelialization capabilities.^119^ A comparison of markers found in MSCs as well as their derived EVs can be found in Table 3.

### 3.4. Meta-analyzing the MSC-EV protein and miRNA landscapes to probe effector functionality

**Fig. 3.**
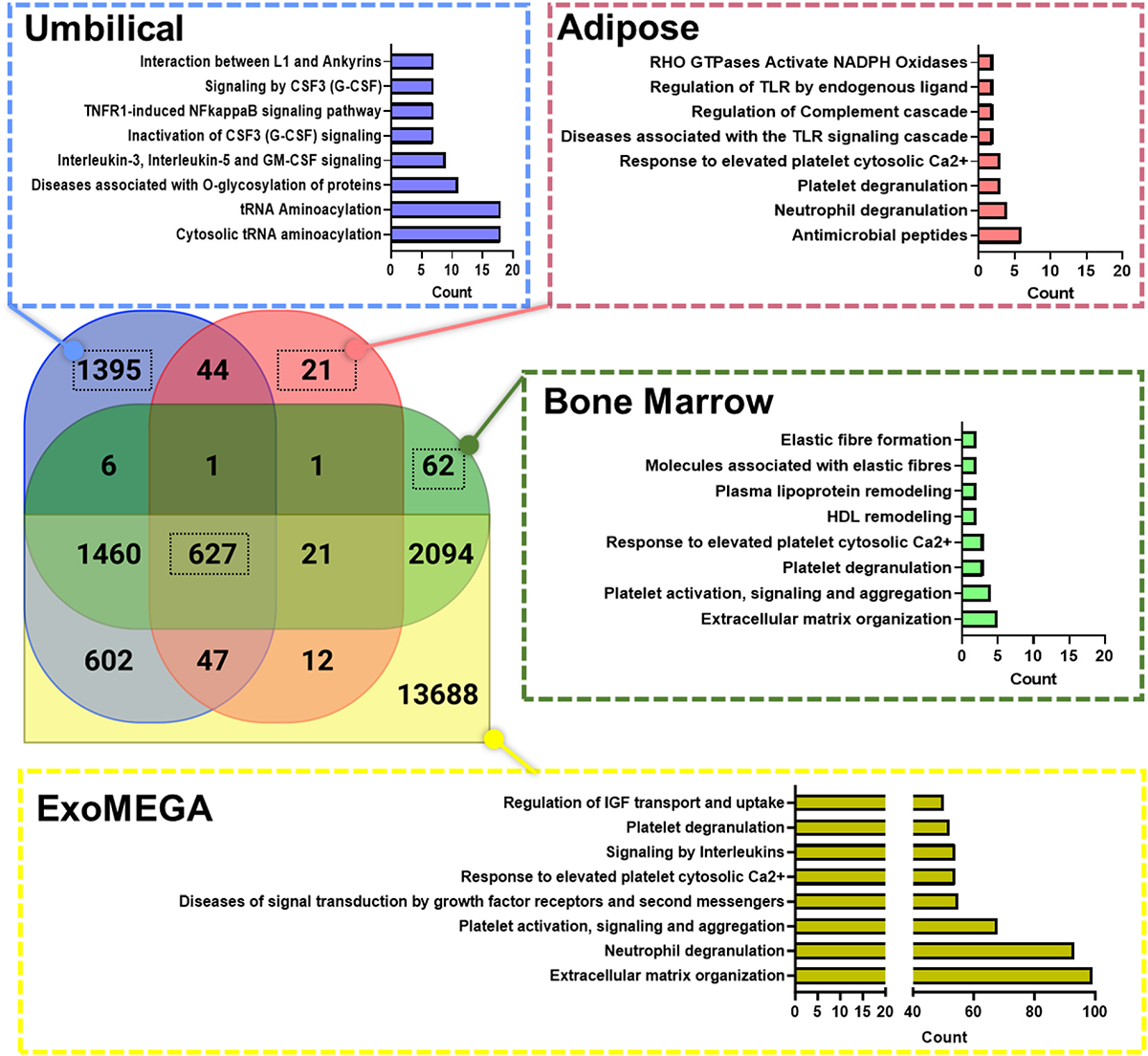
The MSC-EV tissue-specific protein-driven Reactome^140^ ontology analysis of MSC-EVs using the clusterProfiler tool.^135,136^ This analysis aimed to identify the pathways most significantly associated with the examined tissues, adipose,^29,34^ bone marrow,^29,32,141–143^ and umbilical.^29–31,37,144^ We collected articles (N = 10) from the proteome identifications (PRIDE) database using the keywords MSC, extracellular vesicles, exosomes, and mesenchymal stem cells on March 3rd, 2023.

**Fig. 4.**
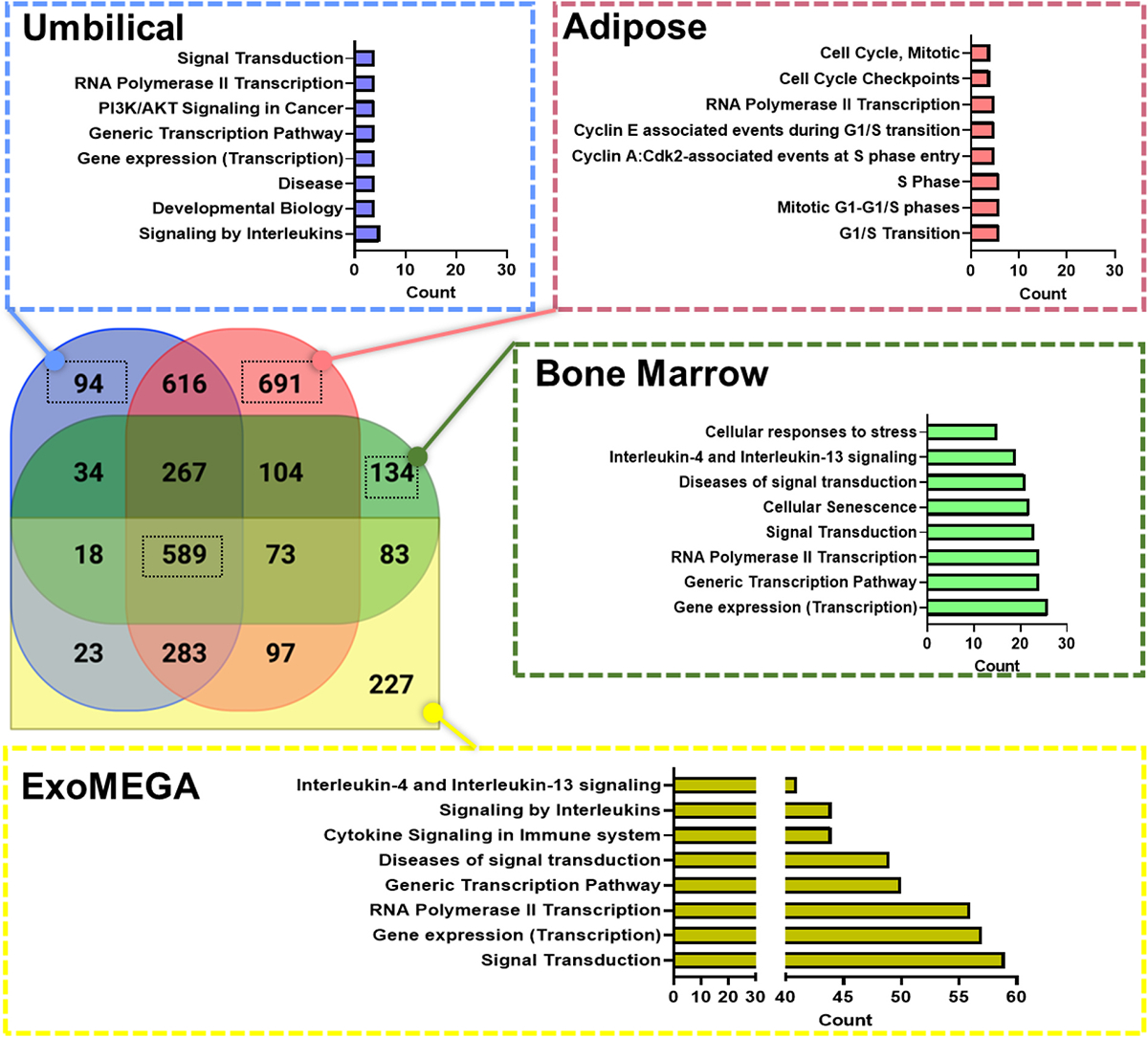
The tissue-specific miRNA-driven Reactome ontology analysis of MSC-EVs using the clusterProfiler tool. This analysis aimed to identify the pathways most significantly associated with the miRNA profiles of the EVs from their source tissue, adipose,^33–35,151,152^ bone marrow,^33,153,154^ and umbilical,^37,151,155–157^ using a significance threshold of p-adj <0.05. We collected articles (N = 10) by curating from Gene Expression Omnibus (GEO) and Sequence Run Archive (SRA) database using the keywords MSC, extracellular vesicles, exosomes, and mesenchymal stem cells on March 3rd, 2023.

Realizing the potential of MSC-EV usage for specific therapeutic results, however, still requires further characterization of EVs not only regarding their molecular markers but also their genomic, proteomic and lipidomic profiles. To achieve more precise molecularly definition of EVs from various MSCs sources, we performed meta-analysis on reported MSC-EV multi-omic datasets as shown in Figs. 3 and 4. Few studies thus far have comprehensively defined the functional landscape of MSC-EVs utilizing available multi-omics datasets. We aimed to address this knowledge gap by conducting a meta-analysis that combines both miRNA and proteomics data to understand better the unique effector functions of MSC-EVs isolated from bone marrow, umbilical cord, and adipose tissue. To gather the necessary MSC-EV datasets, we queried the Proteomics Identifications database (PRIDE)^130,131^ (N = 10) and the Gene Expression Omnibus (GEO)^132–134^ (N = 10). Unfortunately, it was infeasible to standardize the acquired biological profiles through batch correction or reverting the processed datasets to a standardized file format. Therefore, we treated each dataset’s discovered biological profile as-is to apply set analysis in obtaining unique omic profiles relative to each MSCs source for enrichment analysis.

We utilized clusterProfiler^135^ for enrichment analysis to determine the pathways found in the Reactome database.^136^ Reactome focuses on human biology, covering many pathways related to metabolism, signaling, and cellular events. To evaluate the pathways discovered, we utilized the Benjamini-Hochberg^137^ adjusted statistical significance with a threshold of p-adj <0.05, which helps control for false positive results. We were particularly interested in determining whether our meta-analysis would identify protein profiles unfound in curated EV profile databases such as Vesiclepedia^138^ and ExoCarta,^139^ both often used for validation. To address this, we combined Vesiclepedia and ExoCarta to create a super-dictionary database named ExoMEGA. By matching MSC-EV profiles to ExoMEGA, we improved the analytical rigor required to capture a comprehensive omic landscape for MSC-EVs (Figs. 3 and 4). Without mapping to ExoMEGA, the subsequent analyses would be skewed towards the collected datasets, which may not represent the true omic landscape of MSC-EVs. We observed that 84% of the MSC-EV proteins were found in ExoMEGA. From this observation, we applied pathway analysis to reveal distinct top Reactome pathways across adipose, bone marrow, and umbilical EVs (Fig. 3). Fig. 1 shows a list of representative protein and miRNA markers identified in the tissue specific Reactome analysis for adipose, bone marrow and umbilical EVs.

However, uncovering miRNA-associated pathways requires an analysis of known miRNAs that affect target genes, which can then be extrapolated to pathway discovery. To facilitate this analysis, we utilized a widely recognized miRNA-to-gene database called miRPathDB^145^ which has been used in pathway discovery studies for EVs.^146–150^ By leveraging miRPathDB, we translated the miRNA profiles accumulated from each tissue-specific EV type into their corresponding target genes for pathway analysis. As a result of the miRNA analysis, we discovered distinct patterns of top Reactome pathways across adipose, bone marrow, and umbilical EVs. This comparison also revealed a significant proportion of MSC-EV miRNAs not present in the ExoMEGA database (34% found), which may indicate the need for update and expansion of Vesiclepedia and ExoCarta database.

Our pathway analysis revealed that bone marrow EVs primarily impact the tissue microenvironment by remodeling elastic fibers, lipoproteins, the extracellular matrix, and signal transduction pathways. Bone marrow derived EVs displayed a prominent potential role in tissue homeostasis and structural integrity through these modifications. In contrast, umbilical EVs were found to influence the microenvironment through immunomodulation, alteration of gene expression by tRNA aminoacylation, and signal transduction, suggesting their crucial role in regulating immune responses and gene expression patterns within the tissue. Adipose-derived EVs influence the tissue microenvironment by regulating toll-like receptors, degranulation processes, regulation of the Complement cascade and antimicrobial peptide transmission to combat microbial infections and inducing cell proliferation.^158,159^ Our analysis further highlighted the individual uniqueness from different type of MSC-EVs across each tissue, prompting further investigation to elucidate specific molecular mechanisms and cargo molecules, in turn providing deeper insights into their biologic potency and facilitating their development in targeted therapeutic interventions.

Regarding the general function of MSC-EVs, our analysis highlighted that MSC-EVs derived from adipose, bone marrow, and umbilical cord tissues, in alignment with ExoMEGA, exerted influence on various biological processes related to gene expression and transcription, signal transduction, immunoregulation, and tissue regeneration. Broadly, our analysis revealed the involvement of MSC-EVs in tissue regenerative processes. These EVs were generally associated with functions such as platelet degranulation, insulin-like growth factor (IGF) transport, platelet activation, and extracellular matrix (ECM) organization. The regenerative functions shared in common from adipose, bone marrow, and umbilical cord EVs support the therapeutic potential of MSC-EVs in regenerative medicine applications, where they could be harnessed to enhance tissue healing and promote recovery in various disease conditions.

### 3.5. Clinical potential of MSC-EVs

As of December 2023, there are 46 clinical trials registered at ClinicalTrials.gov using MSC-EVs as therapeutics shown in Table 4. The most popular tissue sources are bone marrow (12 trials), umbilical cord (11 trials), and adipose tissue (6 trials). There are 13 trials related to COVID-19, making it the most popular disease to be treated using MSC-EVs during pandemic. It is also worth noting that 11 clinical trials did not specify the tissue source of MSCs, suggesting that perhaps many clinical researchers and physicians are unaware of the variety of MSC-EVs based on origin and tissue sources. As noted above, the listing of trials on this website does not indicate that (if located in the U.S.) they are being conducted under appropriate FDA oversight.

**Fig. 5.**
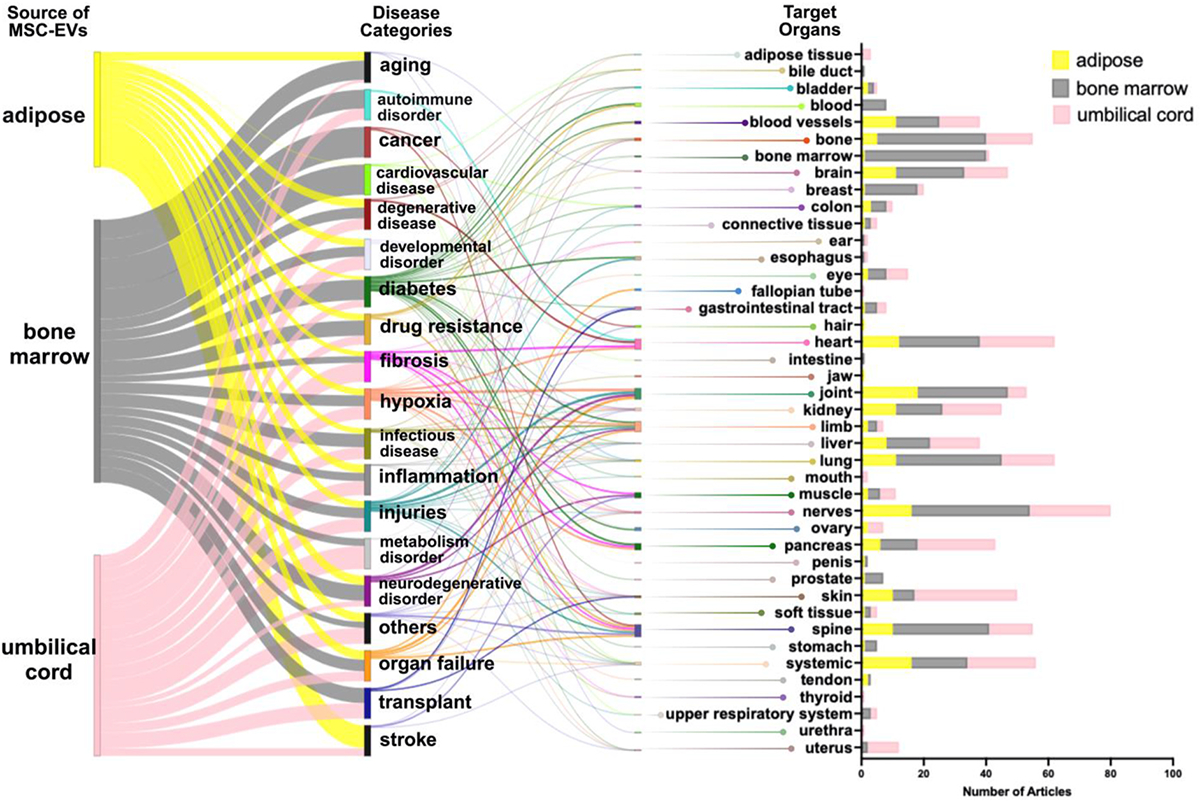
The Sankey visualization comprehensively depicts the impact of MSC-EV isolates as reported in the literature on various disease families and their target organs. Sankey visualization^193^ employs nodes and directed links to represent the flow and distribution of information. Within the diagram, each node corresponds to a distinct subtype within the major groups, while the links demonstrate MSC-EV usage within each group. Notably, the width of these links accurately reflects the frequency of occurrence between different node families, thereby enabling a clear visualization of the relative clinical significance of MSC-EVs across a broad spectrum of diseases and organs. Sankey visualization aids in identifying patterns and trends in the literature, shedding light on the potential therapeutic applications of MSC-EVs and their specific impact on various disease categories and target organs.

Literature search in PubMed with the query “((mesenchymal stem cell) AND (extracellular vesicle)) NOT (review [Publication Type])” returned 3197 results as of April 9th’ 2023. Searches specific to source tissues of MSC-EVs are also performed. The query “(((bone marrow) AND (mesenchymal stem cell)) AND (extracellular vesicle)) NOT (review [Publication Type])” returned 932 results. Queries for adipose and umbilical using the same criteria returned 485 and 568 results, respectively. Those research articles reported a large number of pre-clinical and clinical studies of MSC-EVs involved in a variety of diseases. We extracted the diseases studied in these research articles from their titles and counted the number of articles for each disease with help from ChatGPT, a language learning model published by OpenAI.^160^ We leveraged ChatGPT to “crawl” through each article within our extracted PubMed query to output an initial dataset detailing how MSC-EVs were utilized in each study. Due to inconsistency of the counting results provided by ChatGPT, manual corrections were performed to generate the final dataset. In total, 335 articles for bone marrow, 141 articles for adipose and 241 articles for umbilical cord derived MSC-EV are used for the analysis. As we summarized in Fig. 5, the most studied disease families for MSC-EVs are injury, cancer, degenerative disease, hypoxia and inflammation. In addition, autoimmune disorders, cardiovascular diseases, diabetes and its related complications are also widely studied. An analysis of the targeted organs shows that MSC-EVs are mostly studied for diseases originating in or affecting nerves, lung, brain, kidney, blood vessels, heart, pancreas, skin, bone, joint, liver and spine. As MSC-derived EV have been recognized as important in recent years, many review articles have well summarized therapeutic effects of MSC-EV in various diseases, including autoimmune diseases,^161–163^ cancer,^164^ cardiovascular disease,^165,166^ diabetes,^167,168^ fibrosis,^169^ inflammation,^170–172^ degenerative disease,^173^ and injury.^174^ Other articles reviewed clinical potential of MSC-EV targeting different organs, such as bone,^175^ brain,^176^ joint,^177^ kidney,^178–181^ liver,^182^ lung,^183–186^ nervous system,^187^ skin,^188^ spine,^189^ heart,^190^ and vascular system.^191,192^

However, MSC-EVs from different source tissues are not used identically for some disease families and targeted organs. For example, diabetes and its related complications were mostly studied with MSC-EVs from umbilical cord, but rarely from bone marrow or adipose; in contrary, most studies associated with non-diabetic metabolism disorders were conducted with adipose MSC-EVs; and most of cancer related studies used bone marrow derived MSC-EV. Furthermore, preclinical studies of MSC-EVs for the treatment of diseases tend to choose a source tissue that is closely related to the organ that the disease affects or originates from. Therefore, bone marrow derived MSC-EVs are preferably used for study of diseases related to bone marrow, bone, blood, and spine. Similarly, umbilical cord is a popular MSC-EVs source for diseases of the female reproductive system, including uterus, ovary, and fallopian tube, whereas adipose derived EVs are commonly used to target joints. Moreover, bone marrow is also a preferred source to target the lung and central nervous system, while UC derived MSC-EVs target pancreas and liver diseases. By providing a visual representation of the data, the Sankey diagram facilitates understanding the complex relationships and dynamics involved in using MSC-EVs in different medical contexts, ultimately contributing to advancing research and clinical decision-making in the field.

Applications of MSC-EVs derived from different sources were analyzed through literature and summarized in Fig. 1. The most popular application of BM-MSC-EVs in preclinical study is for bone related diseases. Bone marrow MSC-EVs show therapeutic efficacy toward osteoporosis and osteolysis achieved by cargos including miR-150–3p,^194^ miR-186^195^, miR-150–5p,^196^ MALAT1,^197^ miR-6924–5p.^198^ Spinal cord injury is another disease that is studied for the use of BM-MSC-EVs through regulation of inflammation^199–202^ and apoptosis.^203–205^ As the most popular candidate for cancer related research, BM-MSC-EVs are mostly studied in cancers arisen in the bone marrow. Although some studies found BM-MSC-EVs promote myeloma and myeloid leukemia,^206,207^ others suggest anti-tumoral effects in acute myeloid leukemia (AML)^208^ and colorectal cancer.^209^ Therapeutic effects of AT-MSC derived EV are broadly studied in joint related degenerative diseases, especially osteoarthritis via production of type I and III collagen,^210,211^ regeneration of extracellular matrix (ECM),^212^ and immunomodulation.^213,214^ However, a comparative study between BM and AT-MSC-EVs in an osteoarthritis mouse model indicate that BM-MSC-EVs can better induce type II collagen expression in the knee.^215^ In addition, AT-MSC-EVs can improve metabolic syndrome associated with vascular disease by angiogenesis^216^ and inflammatory regulation.^217,218^ UC-MSC-EVs are notably studied in various diseases associated with the female reproductive system, including polycystic ovary syndrome (PCOS),^219^ intrauterine adhesion (IUA),^218,220^ and thin endometrium.^221^ UC-MSC-EVs are also effective in the treatment of diabetes and its related complications, especially wound healing. Various studies identified islet cell regeneration,^222^ reduced blood glucose levels through liver glycogen storage restoration,^223^ anti-apoptosis in β-cell^224^ as the therapeutic mechanisms for type 2 diabetes. Chronic wound associated with diabetes is also improved by UC-MSC-EVs, highlighting its capability in angiogenesis^99,225–227^ and reduction of inflammatory infiltration.^228^

### 3.6. Future perspective and challenges

As a cell-free therapy, MSC-EVs provide for a more practical supply chain, predictable composition, straightforward handling and therapeutic administration than their parent cells. MSC-EVs have demonstrated biological impacts comparable to their parent cells in past studies, communicating the regenerative and anti-inflammatory properties of MSCs.^229–231^ MSC-EVs maintain a more stable phenotype compared to MSCs’ characteristic differentiation during culture.^232^ Additionally, though MSCs were once thought to be immune-privileged, allogenic MSCs can trigger anti-donor immune responses.^233,234^ In contrast, MSC-EVs appear to be less immunogenic,^231,235^ and accordingly may provide an advantage from the perspective of immune responses that could limit efficacy in the context of repeated dosing. Clinical application of MSC-EVs has been emerging as an alternative allogenic therapy.

The role of MSC-EVs in tumorigenesis is still controversial as some studies found them tumor-suppressive while the others found them tumor-promoting.^164^ Several studies have explored engineered MSC-EVs that carry anti-cancer drugs or miRNAs, and are capable of attenuating tumor growth. The effects of MSC-EVs in various types of cancer has previously been reviewed by Weng et al..^164^ MSC-EVs derived from different tissues have been studied to various extents, and the targeted cancer type also varies. Among all literature that we captured and analyzed, 102 cancer-related studies involving MSC-EVs used bone marrow as the source tissue, whereas only 10 and 6 studies for adipose and umbilical cord, respectively. Most BM-MSC-EVs studies were conducted in osteosarcoma, breast cancer and cancers originating from bone marrow, such as multiple myeloma and myeloid leukemia. Some controversial research reported recently may be due to inconsistent MSCs tissue production and isolation of MSC-EVs. Interestingly, it has been reported that transcriptomic analysis of UC-MSC-EVs from 32 human donors revealed variations in immune modulatory genes which were related to donor-dependent variation in therapeutic efficacy.^236^ The other report found that obesity significantly alters the miRNA expression profile in AT-MSC-EVs, which leads to decreased immunomodulatory and regenerative ability of such EVs.^237^ Accordingly, as would be expected, the tissue sources for derivation of EVs will require control of quality and donor health status. The culture and production pipeline will also require precision quality control to ensure consistent therapeutic utility. Ongoing research will be essential to identify which therapeutic properties of MSC-EVs are shared by EVs from multiple tissue sources and are thus “class effects” of this emerging category of therapeutics; and which other properties are tissue-specific and would thus suggest reasons to select particular MSC-EVs sources or subtypes for clinical translation targeting specific diseases.

## Figures and Tables

**Table 1:** Table of current patents for different types of stem cell culture media.

Patent	Media Type	Description	Ref.
WO2015042356 “Chemically Defined Culture medium for Stem Cell Maintenance and Differentiation”	Chemically Defined	Low protein medium comprising of a volume expander, a lipid mix, and a growth factor modulator.	^81^
**US5908782A** “Chemically Defined Medium for Human Mesenchymal Stem Cells”	Chemically Defined	Composition and method for maintaining human mesenchymal precursor cells in a serum-free environment including (1) a minimum essential medium; (2) serum albumin; (3) an ion source; (4) insulin or an insulin-like growth factor; and (5) at least one amino acid selected from glutamine, arginine, and cysteine.	^82^
**US5045467A** “Serum-Free Growth Medium and Use Thereof”	Serum-Free	Serum-free growth medium comprising an iron-chelate, aurin-tricarboxylic acid and optionally alkali-metal-EDTA and trace elements together with possible growth factors, wherein the iron-chelate may comprise a mixture of Fe-EDTA and citric acid.	^83^
**WO2005113751A1** “Cell Culture Environments for the Serum-Free Expansion of Mesenchymal Stem Cells”	Serum-Free	Compositions and methods for promoting mesenchymal stem cell expansion while maintaining a pluripotent phenotype are disclosed. Serum-free cell culture systems and kits and methods of use for mesenchymal stem cell expansion are provided.	^84^
**WO2015121471A1** “Serum-free medium”	Serum-Free	A serum-free medium for the growth of mesenchymal stem cells comprises FGF, TGF-β and lipoprotein.	^85^

**Table 2:** Characteristics of MSC-EVs cultured in 3D environments.

Culture Method	Cell type	Media	EV size	EV fold production	EV Markers	Findings	Ref
Spheroid	UC-MSCs	Knock-out serum replacement	~110 nm	~9 × 10^10^ particles/mL	CD63, CD9, CD81, Alix, and TSG101	Increase the migration and proliferation of murine fibroblasts *in vitro*	^95^
Spheroid	Placental-MSCs	65% α-MEM, 17% AmnioMAX^™^ C-100 basal media,15% FBS, 2% AmnioMAX^™^ C-100 supplement, 1% GlutaMax), and 2.5 μg/mL Gentamicin	95.6 ± 1.8 nm	From 10^5^ cells, EV yield from 3D culture is 50.3 ± 1.2 μg compared with 2D culture 28.4 ± 1.2 μg	CD9, CD63, CD81	3D culture EV’s protected kidney from progression ischemia-reperfusion (I/R)-AKI and MicroRNA profiling revealed miR-93-p presence	^96^
Microfluidic device/bioreactor	BMSCs	α-MEM, 10% FBS, and 1% penicillin/streptomycin	~180 nm	3D dynamic yield 5.2-fold compared to the static and 2.7-fold compared to the 2D group	Expression of metabolic markers and EV biogenesis markers: STAM1, ALIX, TSG101, HRS and SMPD2, SMPD3, Rab7a, Rab27a, Rab27b, and Rab 31	Dynamic aggregation was found to promote hMSCs exosome/EV production compared to the static aggregate culture	^97^
Bioreactor/dynamic culture	AT-MSCs	PPRF-msc6	~100 nm	Not reported	FLOT1, ICAM, ALIX, CD81, CD63, EpCAM, ANXA5	EV’s derived from dynamic 3D culture upregulated type II collagen production in MSC’s and promoted articular cartilage repair	^98^
Hydrogel	UC-MSCs cocultured HUVECS	DMEM/F12 containing 10% exosome-free serum and 1% penicillin-streptomycin solution	~150 nm	1 × 10^10^ particles/ml	CD63 and CD81	UC-MSC’s derived exosomes cultured in PF-127 promote diabetic wound healing	^99^

**Table 3:** Molecular markers for MSCs and derived EVs as discussed in different studies; “+” signifies positive expression, “-” signifies negative expression. “N/A” -

Marker	Expression	Biological Functionality	MSCs	MSC-EVs
CD105	+	Coreceptor of TGF-beta; associated to angiogenic pathway induction	102,106	103,104
CD11b	−	Regulates cell adhesion, migration, and phagocytosis in immune cells	102,106	103,104
CD14	−	Glycosylphosphatidylinositol-anchored receptor; serves as a co-receptor for toll-like receptors	102,106	103,104
CD146	+	Associated to multipotency, cell migration, vessel formation and angiogenesis	122	N/A
CD173	+	Biosynthetic precursor of the A and B antigens; associated to homing process of immature stem cells to bone marrow	102,106	103,104
CD19	−	Establishes B cell signaling thresholds through modulation of B cell receptor-dependent and independent signaling	102,106	103,104
CD200	+	Regulates immune response	123	N/A
CD271	+	Regulates transition from keratinocyte stem cells to transit-amplifying cells	124,125	N/A
CD29	+	Marker for very late activation Ag integrins on cells	126	103
CD32	−	Cellular response regulation (phagocytosis, cytokine stimulation and endocytic transport), and uptake of immune complexes	102,106	103,104
CD349/FZD-9	+	Encode transmembrane domain proteins that are receptors for Wnt signaling proteins	117–120	121
CD44	+	Associated to cell adhesion, hyaluronate degradation, lymphocyte activation, lymph node homing, myelopoiesis, lymphopoiesis, angiogenesis and cytokine release	126	103,104
CD45	−	Receptor-type protein tyrosine phosphatase; associated to the regulation of T cell function	102,106	103,104
CD73	+	Functions as ecto-5′-nucleotidase and a membrane receptor for extracellular matrix protein	123	103,104
CD79alpha	−	B-cell marker that detects B-cell neoplasms	102,106	103,104
CD9	+	Regulates cell differentiation	126	115
CD90	+	Associated to axon growth, nerve regeneration, T cell activation, apoptosis, inflammation, and wound healing. Functions in inflammation and wound healing by synthesizing growth factors, cytokines and extracellular matrix components to repair damaged tissue	102,106, 123	103,104
HLA-DR	−	Presents peptide antigens to either suppress or elicit T-helper-cell responses	102,106	103,104
ITGA11	+	Associated to regulating myofibroblast differentiation and key phenotypic characteristics	122	N/A
NOTCH3	+	Associated to function and survival of vascular smooth muscle cells	122	N/A
SSEA-4	+	Carbohydrate epitope of glycoproteins	127,128	N/A
W5C5	+	Antibody; utilized for MSCs isolation	129	N/A

**Table 4:** Clinical trials using MSC-EVs as therapeutics.

Source Tissue	NCT Identifier	Conditions	Administration	Phase	Status as of Dec 2023
adipose	NCT04388982	Alzheimer’s disease	nasal drip	1/2	unknown
	NCT04998058	bone loss	bone graft implantation with exosomes	1	not yet recruiting
	NCT04276987	COVID-19	aerosol inhalation	1	completed
	NCT05787288	COVID-19	inhalation	1	recruiting
	NCT04313647	healthy	aerosol inhalation	1	completed
	NCT04544215	pulmonary infection caused by gram-negative bacilli resistant to carbapenems	aerosol inhalation	1/2	recruiting
amniotic fluid	NCT05658094	alopecia	injection	N/A	recruiting
bone marrow	NCT05127122	acute respiratory distress syndrome (ARDS)	IV injection	1/2	not yet recruiting
	NCT03857841	bronchopulmonary dysplasia	IV injection	1	terminated
	NCT05078385	burns	direct application to wound	1	not yet recruiting
	NCT04493242	COVID-19	IV injection	2	completed
	NCT04657458	COVID-19	IV injection	2	active
	NCT05116761	COVID-19	IV injection	1/2	not yet recruiting
	NCT05125562	COVID-19	IV injection	2	withdrawn
	NCT05354141	COVID-19 Acute Respiratory Distress Syndrome	IV injection	3	recruiting
	NCT05130983	Crohn Disease	IV injection	1	recruiting
	NCT04173650	dystrophic epidermolysis bullosa	direct application to wound	1/2	not yet recruiting
	NCT05215288	solid organ transplant rejection	IV injection	1	not yet recruiting
	NCT05176366	ulcerative colitis	IV injection	1	recruiting
placenta	NCT05402748	Fistula Perianal	injection in fistula tract	1/2	recruiting
	NCT05499156	perianal fistula in patients With Crohn’s Disease	injection	1/2	active
	NCT06072794	premature ovarian insufficiency	IV injection	1	recruiting
pluripotent stem cell (PSC) derived MSCs	NCT05738629	dry eye	eye drop	1/2	not yet recruiting
synovial fluid	NCT05261360	degenerative meniscal injury	intra-articular administration of MSCs or MSC-EVs	2	recruiting
umbilical cord	NCT05813379	aging of skin	injection	1/2	recruiting
	NCT04134676	chronic ulcer	topical therapy of conditioned medium	1	completed
	NCT04798716	COVID-19	IV injection	1/2	not yet recruiting
	NCT05387278	COVID-19	IV injection	1	recruiting
	NCT05808400	COVID-19	Inhalation	1	recruiting
	NCT05871463	decompensated liver cirrhosis	unknown	2	recruiting
	NCT02138331	diabetes mellitus type 1	IV injection	2/3	unknown
	NCT04213248	dry eye	eye drop	1/2	recruiting
	NCT04213248	dry eye with cGVHD	eye drop	1/2	recruiting
	NCT03437759	macular holes	intravitreal injection of MSCs or MSC-EVs	1	not yet recruiting
	NCT05413148	retinitis pigmentosa	subtenon injection of MSCs or MSC-EVs	2/3	recruiting
not specified	NCT04602104	acute respiratory distress syndrome	unknown	1/2	unknown
	NCT03384433	cerebrovascular disorders	IV injection of MSC-EVs loaded with miR-124	1/2	unknown
	NCT04491240	COVID-19	aerosol inhalation	1/2	completed
	NCT04602442	COVID-19	aerosol inhalation	2	unknown
	NCT05216562	COVID-19	IV injection	2/3	recruiting
	NCT05243368	cutaneous ulcers	Nutritional supplementation	N/A	not yet recruiting
	NCT05060107	knee osteoarthritis	injection	1	unknown
	NCT04356300	multiple organ dysfunction syndrome (MODS)	IV injection	N/A	not yet recruiting
	NCT05669144	Myocardial Infarction	Intracoronary and intra-myocardial injection of exosomes or mitochondria or both	1/2	recruiting
	NCT05523011	psoriasis	ointment	1	completed
	NCT05520125	segmental fracture - bone loss	surgery with exosomes	1/2	not yet recruiting
